# Hypolipidemic effects of herbal extracts by reduction of adipocyte differentiation, intracellular neutral lipid content, lipolysis, fatty acid exchange and lipid droplet motility

**DOI:** 10.1038/s41598-019-47060-4

**Published:** 2019-07-19

**Authors:** Renate Haselgrübler, Peter Lanzerstorfer, Clemens Röhrl, Flora Stübl, Jonas Schurr, Bettina Schwarzinger, Clemens Schwarzinger, Mario Brameshuber, Stefan Wieser, Stephan M. Winkler, Julian Weghuber

**Affiliations:** 1 University of Applied Sciences Upper Austria, Wels, Austria; 2 Institute of Medical Chemistry, Center for Pathobiochemistry and Genetics, Medical University of Vienna, Vienna, Austria; 3 University of Applied Sciences Upper Austria, Hagenberg, Austria; 4 Austrian Competence Center for Feed and Food Quality, Safety and Innovation, Wels, Austria; 5 Johannes Kepler University, Institute for Chemical Technology of Organic Materials, Linz, Austria; 6 Institute of Applied Physics, TU Wien, Vienna, Austria; 7 ICFO-Institut de Ciencies Fotoniques, The Barcelona Institute of Science and Technology, 08860 Castelldefels, Barcelona, Spain

**Keywords:** Glycobiology, Lipidomics, Glycerides

## Abstract

An increase in adipose tissue is caused by the increased size and number of adipocytes. Lipids accumulate in intracellular stores, known as lipid droplets (LDs). Recent studies suggest that parameters such as LD size, shape and dynamics are closely related to the development of obesity. Berberine (BBR), a natural plant alkaloid, has been demonstrated to possess anti-obesity effects. However, it remains unknown which cellular processes are affected by this compound or how effective herbal extracts containing BBR and other alkaloids actually are. For this study, we used extracts of *Coptis chinensis*, *Mahonia aquifolium*, *Berberis vulgaris* and *Chelidonium majus* containing BBR and other alkaloids and studied various processes related to adipocyte functionality. The presence of extracts resulted in reduced adipocyte differentiation, as well as neutral lipid content and rate of lipolysis. We observed that the intracellular fatty acid exchange was reduced in different LD size fractions upon treatment with BBR and *Coptis chinensis*. In addition, LD motility was decreased upon incubation with BBR, *Coptis chinensis* and *Chelidonium majus* extracts. Furthermore, *Chelidonium majus* was identified as a potent fatty acid uptake inhibitor. This is the first study that demonstrates the selected regulatory effects of herbal extracts on adipocyte function.

## Introduction

Obesity is a complex, chronic disorder caused by the interaction of different contributing parameters, including dietary, lifestyle, genetic, and environmental factors. Appropriate lifestyle and behavioral interventions are the fundamentals of weight loss success; however, maintaining such a healthy lifestyle is often challenging for many people. Several pharmacological strategies have been tested to manage obesity over the years. However, many of the anti-obesity drugs that were approved and marketed have now been withdrawn due to serious adverse effects, such as depression, anxiety, and elevated cardiovascular risk^1^. Therefore, dietary phytogenic substances might be applied as alternatives to synthetic anti-obesity agents, with little or no toxic side effects.

Obesity is associated with an increase in adipose tissue, which is caused not only by increased adipocyte size (hypertrophy) but also by increased adipocyte number (hyperplasia). Hyperplasia is regulated by the *de novo* differentiation of preadipocytes, which are located in the stromal-vascular fraction of adipose tissue^2–4^. Therefore, the regulation of adipocyte differentiation might be of pivotal importance for the prevention and treatment of obesity and its related diseases.

In this regard, the natural isoquinoline alkaloid berberine (BBR) has been reported to have great potential. BBR can be found in various medicinal plants, such as *Hydrastis canadensis*, *Coptis chinensis*, *Berberis aquifolium*, *Berberis vulgaris* and *Berberis aristata*^5^, and has been demonstrated to reduce body weight, blood glucose and lipid levels in experimental and clinical studies, suggesting the potential for use as a hypolipidemic drug^6–10^. The anti-obesity effects of BBR have been attributed to its inhibition of adipocyte differentiation, which is primarily caused by the reduced expression of important regulatory adipogenic transcription factors, enzymes and receptors, such as (i) sterol regulatory element-binding proteins (SREBPs)^11–13^, (ii) fatty acid synthase (FAS)^11,12^, (iii) peroxisome proliferator-activated receptor-gamma (PPARγ)^12,14^, and (iv) CCAAT/enhancer binding proteins (C/EBPs)^14^. In addition, BBR exerts cholesterol-lowering effects by stabilizing LDLR (low-density lipoprotein receptor) mRNA and protein^7,15^.

Intracellular lipid storage primarily occurs in lipid droplets (LDs). LDs have been considered to be static and inert energy depots for many years; however, LDs have recently been redefined as active organelles involved in cellular metabolism^16–18^. Therefore, the role of LDs in the development of obesity and related diseases might be currently underestimated, as the amount of intracellular lipid and the way that lipids are processed and stored are both of importance^19^. Recent developments in microscopy and spectroscopy have introduced new possibilities for examining intracellular lipids^16^. It is now obvious that LD shape, size, intracellular localization and protein coatings can affect LD dynamics and, thus, correlate with the development of metabolic disorders^19–21^. However, the regulatory role of phytogenic substances in general, and especially of BBR in particular, remains to be elucidated in this context.

In the present study, we used two different cell models to investigate the potential hypolipidemic effects of herbal extracts: (i) 3T3-L1 cells, which can be differentiated into an adipocyte-like phenotype and are therefore a widely used *in vitro* model of white adipocytes^22^, and (ii) HuH7 cells (human hepatocytes), as this cell line has been reported to contain a high number of LDs of an average intermediate size^23^. Here, we showed that BBR and potential alkaloid-enriched herbal extracts can reduce the following: (i) adipocyte differentiation, (ii) the content of intracellular neutral lipids and cholesterol, (iii) lipolysis, (iv) fatty acid uptake, (v) intracellular lipid exchange in LDs and vi) LD motility.

## Materials and Methods

### DNA constructs and reagents

The ADRP-GFP plasmid was kindly provided by John MacLauchlan (University of Glasgow, Glasgow, Scotland). Insulin from bovine pancreas, dexamethasone, 3-isobutyl-1-methylxanthine (IBMX) and berberine (BBR) were purchased from Sigma-Aldrich (Schnelldorf, Germany). LD540 was a kind gift from Christoph Thiele (University of Bonn, Bonn, Germany). Herbal extracts were obtained from The Plant Extract Collection Kiel in Schleswig-Holstein (PECKISH), an open access screening library^24^, which we have successfully used for screening applications^25,26^. For this study, water extracts from Chinese goldthread (*Coptis chinensis rad*., PECKISH number #3079), barberry (*Berberis vulgaris cortex lign*., #0193), opium poppy (*Papaver somniferum capsula*, #1808), mahonia (*Mahonia aquifolium rad*., #2457) and tetterwort (*Chelidonium majus herb*., #2927) were used. For cell culture experiments, extracts were diluted in the respective cell culture media.

### Cell culture

HuH7 and 3T3-L1 cells were purchased from ATCC (Manassas, USA). Both cell lines were maintained in Dulbecco’s modified Eagle’s medium (DMEM), supplemented with 100 μg/mL penicillin, 100 μg/L streptomycin, and 10% FBS (all Biochrom GmbH, Berlin, Germany; medium 1), and grown in a humidified atmosphere at 37 °C and 5% CO_2_. The differentiation of the 3T3-L1 cells was initiated 2 days post-confluence by the addition of 0.25 µM dexamethasone, 500 µM 3-isobutyl-1-methylxanthine (IBMX) and 10 µg/mL insulin (termed medium 2) and allowed to proceed for 3–4 days. The differentiation medium was subsequently replaced with complete growth medium supplemented with 10 µg/mL insulin (termed medium 3) and cells were allowed to proceed for further 7–10 days. For the generation of HuH7 cells stably expressing ADRP-GFP, cells were transfected using Lipofectamine LTX Reagent (Thermo Fisher Scientific, Waltham, Massachusetts, USA), according to the manufacturer’s protocol. Cells were plated into 60 mm culture dishes and grown for 48 hours. The medium was removed and replaced by medium supplemented with 400 µg/mL G418 (Biochrom GmbH, Berlin, Germany). This medium was changed every 3 days, and 15–20 days later, individual G418-resistant colonies were selected for propagation and analysis.

### LD540 uptake study

3T3-L1 cells were grown and differentiated in 12-well tissue culture plates. Cells were treated for 1 or 3 days with the indicated substances (BBR: 3.70 µg/mL, herbal extracts: 10 mg/L) post-differentiation or left untreated (control cells). Cells were subsequently pulse labeled with LD540 (0.5 µg/mL) for 20 sec, washed once with medium and further incubated in LD540-free medium for 30 min. After the transport period, cells were washed with PBS twice and then lysed in 0.05 M NaOH. A volume of 300 µL of the lysate was transferred into a 96-well plate, and total fluorescence was quantitated with a microplate reader (544 nm excitation, 590 nm emission; POLARstar Omega, BMG LABTECH, Ortenberg, Germany). Data were analyzed using the Omega MARS Data analysis software package (BMG LABTECH, Ortenberg, Germany). The LD540 uptake was normalized to untreated cells grown under the same conditions. The same experimental procedure was used for studying LD540 uptake in HuH7 cells, except for the differentiation step.

### Nile red staining

Neutral lipid accumulation in 3T3-L1 cells was assessed by the lipophilic dye Nile red (Thermo Fisher Scientific, Waltham, Massachusetts). Cells were treated with the indicated substances (BBR: 3.70 µg/mL, herbal extracts: 10 mg/L) for different time periods, washed with PBS and fixed with 4% paraformaldehyde for 15 min. The cells were washed three times with PBS and were subsequently stained with 10 µg/mL Nile red solution for 15 min. Stained cells were rinsed twice with PBS, and LDs were imaged on an Olympus IX-81 inverted microscope (Olympus, Tokyo, Japan), equipped with an IX2-DSU confocal unit. Nile red quantitation was performed using a microplate reader (544 nm excitation, 590 nm emission; POLARstar Omega, BMG LABTECH, Ortenberg, Germany). Data were analyzed using the Omega MARS Data analysis software package (BMG LABTECH, Ortenberg, Germany). Nile red staining was normalized to untreated cells grown under the same conditions.

### Measurement of glycerol release

Differentiated 3T3-L1 adipocytes in 96-well plates were preincubated in insulin-free medium 24 hours before lipolysis experiments. Cells were treated for 24 hours with the indicated substances (BBR: 3.70 µg/mL, herbal extracts: 10 mg/L) followed by additional induction of lipolysis using 100 nM isoproterenol for 1 hour. Glycerol content in the incubation medium was used as an index for lipolysis and was measured using a lipolysis colorimetric assay kit (Sigma-Aldrich, Schnelldorf, Germany) according to manufacturer’s instruction.

### Live-cell tubulin staining

HuH7 cells stably expressing ADRP-GFP were grown in 96-well imaging plates and stained with SiR-tubulin (1 µM; tebu-bio GmbH, Offenbach, Germany) for 45 min, according to the manufacturer’s protocol. Cells were washed, mounted and imaged on an Olympus IX-81 inverted microscope (Olympus, Tokyo, Japan) equipped with an IX2-DSU confocal unit.

### FRAP experiments

Differentiated 3T3-L1 adipocytes were treated for 1 and 3 days with the indicated substances (BBR: 3.70 µg/mL; herbal extracts: 10 mg/L) or left untreated. Cells were then stained with LD540 (0.5 µg/mL) for 5 min, washed twice with PBS and incubated for 40–60 min in label-free medium. This step ensured that fluorescence reached a steady-state distribution in the cell. Cells were imaged on an Olympus IX-81 inverted microscope (Olympus, Tokyo, Japan) equipped with an IX2-DSU confocal unit. A light guide-coupled illumination system (Olympus U-HGLGPS) with appropriate filters was used to image LD540 fluorescence. The fluorescence signal was recorded by an Orca EM-CCD camera (Hamamatsu Photonics, Herrsching, Germany). For FRAP experiments, single LDs were photobleached with an intense laser pulse (405 nm) applied for 1,000 ms. Recovery images were recorded at the indicated time intervals. FRAP images were initially analyzed using the built-in FRAP module of the Xcellence RT software. Data were normalized with the pre-bleached image and curve fitting was performed using GraphPad Prism software (version 7). The resulting FRAP curves were plotted based on the standard error of the mean (SEM) and fitted using a mono-exponential equation. The kinetic FRAP parameters were directly obtained from curve fitting.

### LD motility analysis

HuH7 cells stably expressing ADRP-GFP were seeded into 96 well imaging plates (5,000 cells/well) and grown to 90% confluency. Cells were subsequently treated with BBR (3.70 µg/mL) or herbal extracts (10 mg/L) for 1 and 3 days or left untreated (control). Nocodazole was used as a negative control. LD tracking was performed on an Olympus IX-81 inverted microscope (Olympus, Tokyo, Japan) in objective-type TIR configuration via an Olympus 60x NA = 1.49 Plan-Apochromat objective as described previously^27,28^. The 96-well plates were placed on an x-y-stage (CMR-STG-MHIX2-motorized table; Märzhäuser, Wetzlar, Germany), and the 488 nm emission of a diode laser (Toptica Photonics, Munich, Germany) was used to image GFP fluorescence. After appropriate filtering, the fluorescence signal was recorded via an Orca EM-CCD camera (Hamamatsu Photonics, Herrsching, Germany). The TIR configuration was chosen to reduce the cytosolic background and to obtain round-shaped fluorescence signals from the bottom of the detected LDs in the evanescent field. Images were taken every 200 ms for a total of 10 seconds. Images were then exported as TIF files, and motility analysis was performed using the Spotty framework (see “Data analysis” for detailed information). For each particle, the position at each time point was determined as the center of the mass of the recorded photon distribution. Subsequently, for each particle, the trajectory over the whole recorded time period was constructed using a nearest distance threshold. These trajectories were then characterized by mean square displacement (MSD) analyses, as previously reported^29^. In short, MSD for different time-lags n*t_lag_ were calculated using the formula MSD_n_ = <(x_i+n_ − x_i_)² + (y_i+n_ − y_i_)²>_i=0…N-1_, where N is the trajectory length, n is the dimensionless time-lag, and x_i_ and y_i_ are the X- and Y-coordinates of the LD in frame I, respectively. The mode of lateral motility was determined by the shape of the MSD curve via visual inspection, where the MSD curve of the pure diffusive mode appears as a straight line, the directed (super-diffusive) mode appears as a progressive curve, and the anomalous (sub-diffusive) mode appears as a degressive curve^30^. A linear fit (MSD = 4Dt_lag_ + 4dx²)) allows the qualification of the segment (set to 0–3 sec) assumed to be pure Brownian motion, and a degressive dependence (MSD = 4Dt_lag_^α^ + 4dx²) of the MSD indicates sub-diffusive motion (0–10 sec). From these fits, the diffusion coefficients (D) and the value of α (anomalous diffusion exponent; characterizes the motion that the LD experiences; for free Brownian motion, α = 1, and for confined motion, 0 < α < 1) were obtained.

### Cytotoxicity assay

The cytotoxic effects of herbal extracts used in this study were evaluated by using a resazurin-based *in vitro* toxicology assay (Sigma-Aldrich, Schnelldorf, Germany), according to the manufacturer’s instructions. Briefly, HuH7 and undifferentiated 3T3-L1 cells were seeded into 96-well plates (45,000 cells/well), grown to 90% confluency, and incubated with the test substances (BBR: 3.70 µg/mL; herbal extracts: 10 mg/L) for 1, 3 and 5 days at 37 °C. Subsequently, the cells were washed with medium and incubated with a medium containing 10% resazurin for 2 h. The concentrations of the reduced form of resazurin (resorufin) were then determined using a microplate reader in fluorescence mode (544 nm excitation, 590 nm emission; POLARstar Omega, BMG LABTECH, Ortenberg, Germany). Data were analyzed using the Omega MARS Data analysis software package (BMG LABTECH, Ortenberg, Germany). Cell viability was normalized to untreated cells grown under the same conditions. Each test substance was measured in triplicate. The same experiment was performed with differentiated 3T3-L1 adipocytes, as well as during differentiation (treatment started at medium 2 addition).

### High pressure liquid chromatography with mass spectrometry

The quantitative analysis of the BBR content of the herbal extracts was performed as previously reported, with minor modifications^31^. Briefly, HPLC analysis was performed using a Thermo Scientific Dionex Ultimate 3000 comprised of an LPG-3400SD pump with a built-in degasser, a WPS-3000 U(T)SL cooled autosampler, a temperature-controlled column compartment and an FLD-34000RS diode array detector (DAD) equipped with Chromeleon software. Analyte separation was performed on an Accucore C18 column (150 mm × 3.0 mm inner diameter, 2.6 µm particle size; Thermo Scientific). The column temperature was set to 40 °C, and the injection volume was 1 µL. UV wavelengths were detected at 260 nm and 360 nm. Analytes were separated by gradient elution, with mobile phase A containing 0.1% formic acid (FA) in water and mobile phase B containing 0.1% FA in acetonitrile, at a flow rate of 0.5 mL/min. The elution gradient starting conditions were 95% A and 5% B. After 5 min of equilibration time, the proportion of B was increased to 20% at 8 min, to 40% at 12 min, to 60% at 15 min and to 80% at 17 min. The proportion of B was reduced to 5% from 20 min to 25 min. BBR content was quantitated against a known standard in a linear range of 0.025–0.5 mg/mL.

The identification of further isoquinoline alkaloids was performed using a Thermo Scientific Surveyor HPLC system coupled to a LTQ Orbitrap Velos. HPLC column and gradient were identical to the conditions described for quantitative HPLC. Mass spectra were recorded with an electrospray ionization interface in FT mode with a resolution of 60000. MS^2^ spectra of the most intense ions were automatically recorded on the LTQ ion trap with collision induced dissociation utilizing a collision energy of 35%. %. Identification of berberine and palmatine is based on comparison with authentic standards, other compounds are tentatively identified based on high resolution m/z values and available literature data on MS^2,32–34^. The berberine:palmatine ratio was calculated by averaging the mass spectra of the complete overlapping peaks and determining the ratio of the molecular ions. Peaks only visible in MS have not been assigned.

### Gas chromatography

Gas chromatography was used to directly quantitate the cellular content of triglycerides, total cholesterol, free cholesterol and cholesterol esters within a single analysis^35,36^. HuH7 cells were grown in 6 cm culture dishes and were allowed to reach confluency. After 1 day post-confluence, cells were treated with the indicated substances for 5 days. 3T3-L1 cells were grown in 6-well cell culture plates and treated with the indicated substances during differentiation (starting point was the addition of differentiation medium 2). After treatment, cells were washed with PBS, detached using trypsin, and lipids were isolated from cell pellets by standard Folch extraction. The cell protein content was measured using the Bradford assay from an aliquot of the cell pellet. Lipids were separated using a GC-2010 gas chromatograph (Shimadzu, Kyoto, Japan) equipped with a programmed temperature vaporizer injector and a ZB-5HT capillary column (15 m × 0.32 mm × 0.1 µm; Phenomenex, Aschaffenburg, Germany). Tri-decanoyl glycerol, cholesteryl myristate and tri-nonadecanoyl glycerol (Sigma-Aldrich) were used as standards for free and esterified cholesterol and triglycerides, respectively. The chromatograms were quantitated using GC solutions 2.3 (Shimadzu), and the results were normalized to cell protein content. Total cholesterol was calculated as the sum of free and esterified cholesterol.

### Data analysis

Initial imaging recordings were supported by the Olympus Xcellence RT software. In-depth analyses for the calculation of the fluorescence intensities in individual cells and a fast comparison of the fluorescent signals in numerous cells at different time intervals was performed using the Spotty framework^37^. Spotty can be retrieved online at http://bioinformatics.fh-hagenberg.at/projects/microprot/. The most important analysis algorithms integrated in the intensity analysis in Spotty are: (a) preprocessing (including correlation based optimal downsampling, filtering and the creation of layer-based images), (b) cell detection, and (c) results analysis. Statistical analysis was performed using a one-way ANOVA followed by a Dunnett’s multiple comparisons test in GraphPad Prism software (version 7). Figures were prepared using Corel Draw (version X6).

## Results

### HPLC analysis

**Figure 1:**
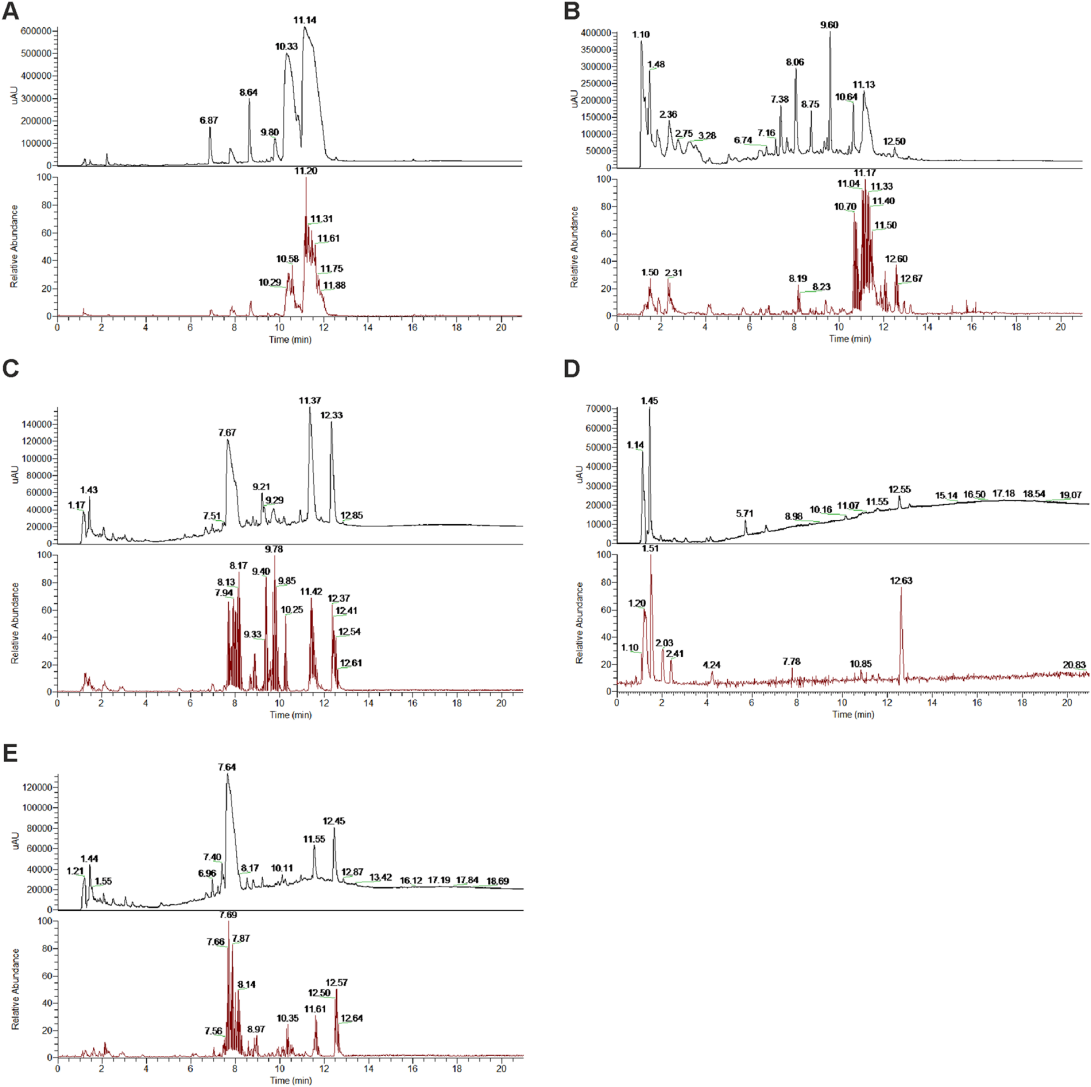
HPLC-DAD and HPLC-MS chromatogram of *C. chinensis* (**A**), *C. majus* (**B**), *M. aquifolium* (**C**), *P. somniferum* (**D**), *B. vulgaris* (**E**).

BBR, a naturally occurring alkaloid, has been shown to reduce lipogenesis *in vitro* and *in vivo* through different mechanisms^38^. We searched the PECKISH library^24^ for extracts that potentially contain BBR and selected water extracts from Chinese goldthread (*C. chinensis*), berberis (*B. vulgaris*), opium poppy (*P. somniferum*), mahonia (*M. aquifolium*) and tetterwort (*C. majus*) for further investigation. HPLC analysis was performed for BBR quantitation prior to *in vitro* cell culture experiments (Fig. 1 and Table 1). Interestingly, the BBR contents exhibited large variations, and BBR was found in extracts from *C. chinensis* (2.78 g/L), *M. aquifolium* (0.03 g/L) and *B. vulgaris* (0.02 g/L). For extracts of *P. somniferum* and *C. majus*, BBR was detected but below the limit of quantitation. However, these extracts were also included in subsequent experiments to investigate the role of other anti-adipogenic phytochemicals. In addition to BBR, we further characterized the extracts under study to identify their main isoquinoline alkaloids using HPLC-MS. Up to nine different lead alkaloids could be clearly identified in the different extracts such as magnoflorine, coptisine, morphine, chelidonine and BBR as well as the BBR-derivatives berberrubine, epiberberine, jatrorrhizine, and palmatine. Representative HPLC-DAD as well as HPLC-MS diagrams indicating the retention times are shown in Fig. 1 for (A) *C. chinensis*, (B) *C. majus*, (C) *M. aquifolium*, (D) *P. somniferum* and (E) *B. vulgaris*. Table 1 summarizes the identified alkaloids present in the individual extracts. For subsequent experiments, cells were either treated with 3.70 µg/mL BBR standard or 10 mg/L extract solution, which accounts for final BBR concentrations of 2.78 µg/mL (*C. chinensis*), 0.03 µg/mL (*C. majus*) and 0.02 µg/mL (*B. vulgaris*) in cell culture experiments.

**Table 1:** Identification of isoquinoline alkaloids of extracts under study using HPLC with DAD and Orbitrap MS.

Extract	Compound	Retention time t_R_ [min]	MS [m/z]	MS^2^ [m/z]
*C. chinensis*	unknown	6.87	—	—
	Magnoflorine	7.78	342.1711	297, 265
	unknown	8.64	—	—
	Berberrubine	9.80	322.1071	307, 294
	Coptisine	10.33	320.0914	305, 292, 290, 262
	Epiberberine	10.33	336.1227	321, 308, 292
	Jatrorrhizine	10.33	338.1383	323, 322, 294
	Berberine	11.14	336.1231	321, 292
	Palmatine	11.14	352.1543	337, 308
*C. majus*	Magnoflorine	8.06	342.1700	297, 265
	unknown	8.75	303.0499	—
	unknown	9.60	303.0499	—
	C_20_H_20_NO_5_, e.g. Chelidonine	10.64	354.1329	—
	Coptisine	11.13	320.0916	305, 292, 290, 262
	C_19_H_18_NO_5_	11.99	340.1185	—
	Berberine	12.50	336.1230	321, 292
	Palmatine	12.50	352.1539	337, 308
*M. aquifolium*	Magnoflorine	7.67	342.1704	297, 265
	unknown	7.99	298.1450	—
	unknown	9.21	298.1445	—
	unknown	9.73	305.1523	—
	unknown	10.19	305.1522	—
	Jatrorrhizine	11.37	338.1398	323, 322, 294
	Berberine	12.33	336.1246	321, 292
	Palmatine	12.33	352.1555	337, 308
*P. somniferum*	unknown	1.45	136.0617	—
	unknown	1.45	152.0565	—
	Morphine	1.94	286.1440	268, 229
	unknown	5.71	—	—
	Berberine	12.55	336.1237	321, 292
	Palmatine	12.55	352.1549	337, 308
*B. vulgaris*	unknown	2.06	192.1021	—
	unknown	2.85	190.0867	—
	unknown	6.96	356.1510	—
	unknown	7.22	358.1667	—
	unknown	7.40	354.1354	—
	Magnoflorine	7.64	342.1707	297, 265
	C_20_H_22_NO_4_	8.52	340.1565	325, 308, 295
	unknown	8.80	314.1764	—
	unknown	10.11	305.1533	—
	unknown	10.11	321.1477	—
	Jatrorrhizine	11.55	338.1394	323, 322, 294
	Berberine	12.45	336.1246	321, 292
	Palmatine	12.45	352.1555	337, 308

Berberine:palmitine ratio for *C. chinensis* (100:60), *C. majus* (100:5), *M. aquifolium* (100:35), *P. somniferum* (100:10), and *B. vulgaris* (100:25).

### Effects of BBR and herbal extracts on HuH7 and 3T3-L1 cell viability

**Figure 2:**
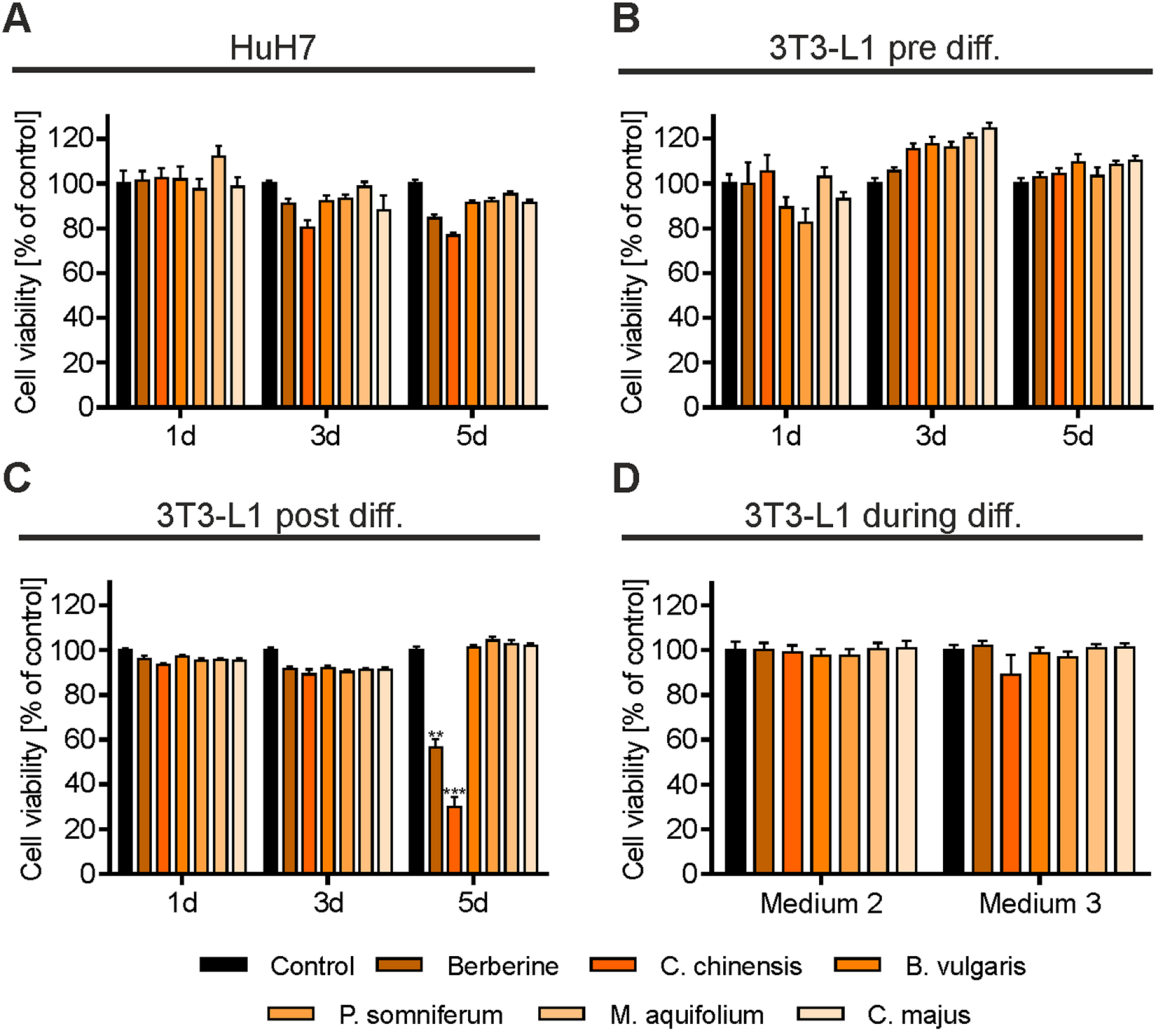
Cytotoxicity of BBR and herbal extracts. Cells were grown in 96 well plates and treated with the indicated substances for 1, 3 and 5 days or during differentiation. Cell viability was measured using a resazurin-based *in vitro* toxicology assay. (**A**) HuH7 cells, (**B**) undifferentiated 3T3-L1 cells, (**C**) differentiated 3T3-L1 cells and (**D**) 3T3-L1 cells treated during the differentiation process. Error bars are based on the SEM of 3 independent experiments.

The cytotoxic effects of BBR and herbal extracts were examined. HuH7, 3T3-L1 pre-adipocytes and differentiated 3T3-L1 cells were treated with BBR and herbal extracts for 1, 3 and 5 days (Fig. 2A–C) or during the differentiation process for 3T3-L1 cells (Fig. 2D). Neither BBR nor the herbal extracts reduced the cell viability of HuH7 or 3T3-L1 pre-adipocytes after 1, 3 and 5 days of treatment. In contrast, BBR (p < 0.01) and *C. chinensis* (p < 0.001) remarkably reduced the cell viability of differentiated 3T3-L1 cells after 5 days of incubation.

### Effects of BBR and herbal extracts on adipogenic differentiation

**Figure 3:**
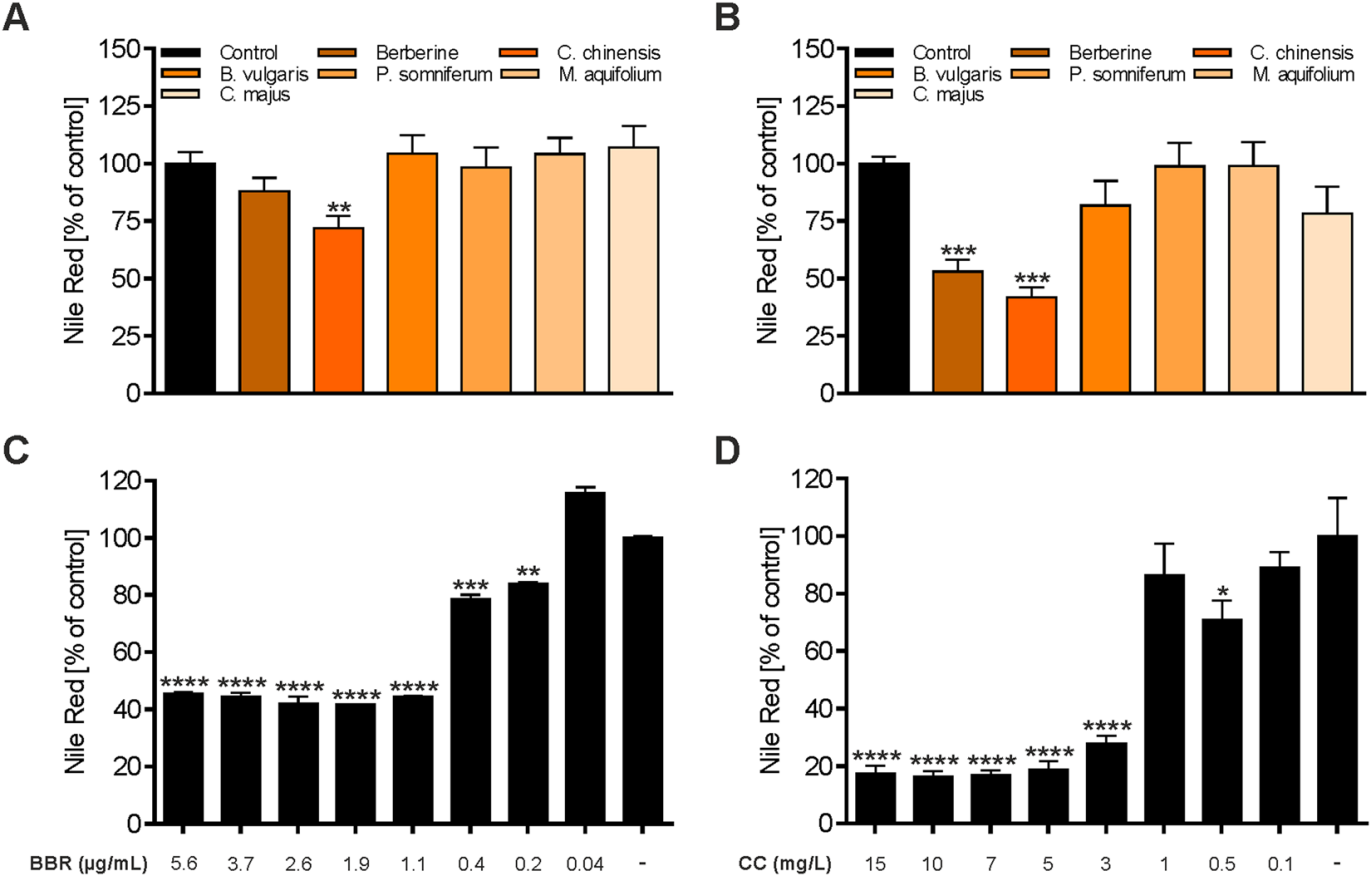
Effects of BBR and herbal extracts on the adipogenesis of 3T3-L1 adipocytes. 3T3-L1 cells were differentiated through incubation with a differentiation cocktail (medium 2 containing dexamethasone, IBMX and insulin mixture), followed by an additional growth phase in dexamethasone- and IBMX-free medium (medium 3 containing insulin). (**A**) Addition of BBR and herbal extracts in medium 3 during the post-differentiation phase. (**B**) Addition of BBR and herbal extracts in medium 2 during differentiation. (**C**) BBR treatment during differentiation with various concentrations. (**D**) *C. chinensis (CC)* treatment during differentiation with various concentrations. Cells were fixed with 4% paraformaldehyde and stained with Nile red at day 10–12 after the start of differentiation. Lipid accumulation was assessed by measuring the fluorescence intensity on a plate reader. Error bars are based on the SEM of at least three independent experiments. *p < 0.05, **p < 0.01, ***p < 0.001 and ****p < 0.0001, significantly reduced Nile red signal of cells treated with the indicated substances compared to untreated control cells.

**Figure 4:**
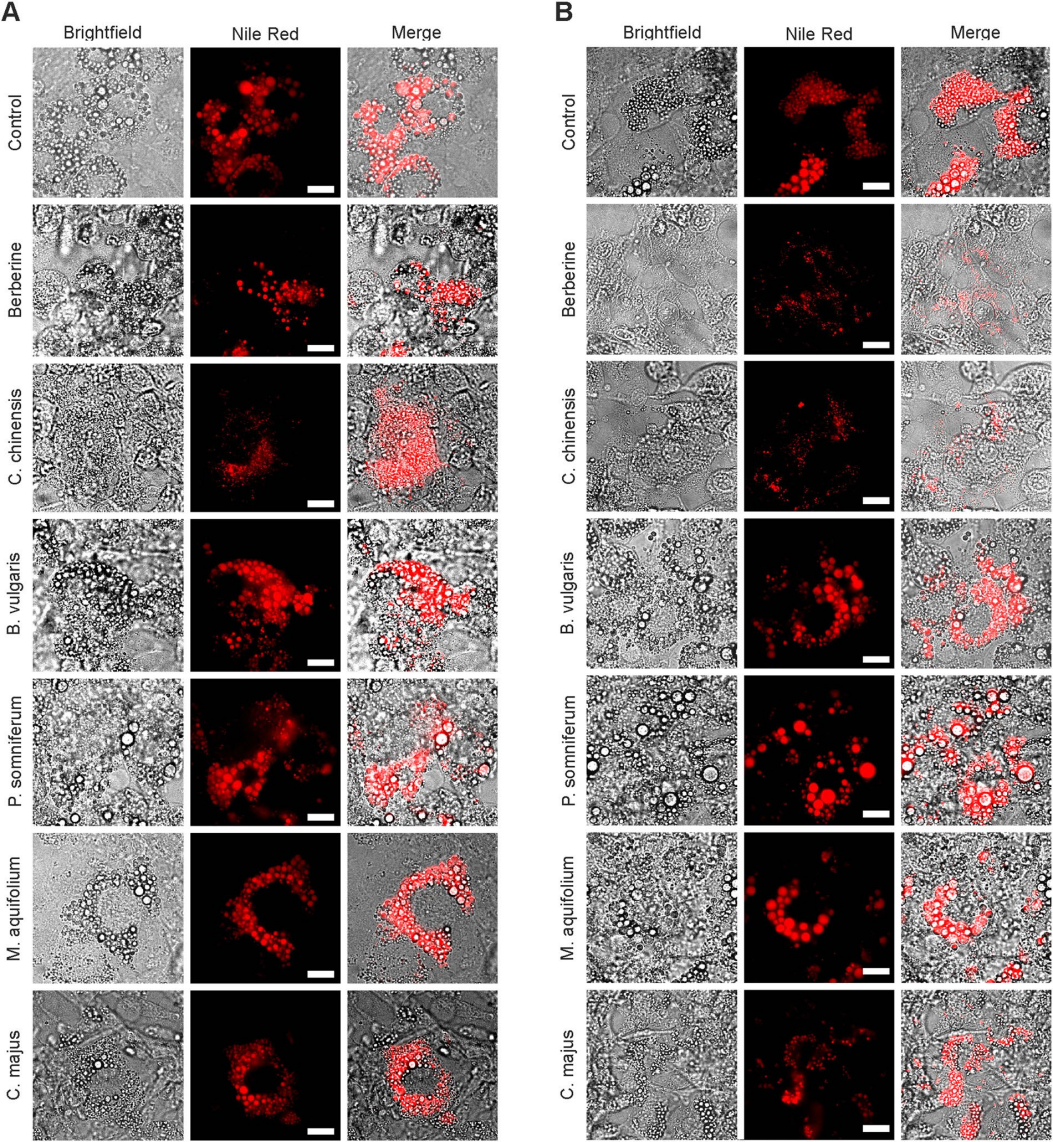
Representative microscopic images of Nile red stained cells treated post-differentiation (in medium 3) (**A**) or during differentiation (in medium 2) (**B**) with the indicated substances. Scale bar = 20 µm.

BBR has been reported to possess inhibitory effects on adipogenesis in 3T3-L1 cells by various mechanisms^10,12,39,40^. To investigate the anti-adipogenic effects of BBR-enriched, as well as BBR-reduced herbal extracts, the intracellular lipid accumulation was determined in pre-adipocytes and in mature 3T3-L1 cells by Nile red staining (Figs 3 and 4). When BBR or herbal extracts were added to the cell culture medium throughout the process of differentiation, 3T3-L1 adipocyte differentiation was significantly inhibited by BBR (p < 0.001) *and C. chinensis* (p < 0.001) compared to untreated cells, as assessed by the quantitation of Nile red fluorescence (Fig. 3B). On the contrary, treatment with *B. vulgaris, C. majus, P. somniferum* and *M. aquifolium* did not lead to a significant inhibition. Based on microscopic observations, we found that 3T3-L1 cells treated with BBR and *C. chinensis* extract maintained the fibroblastic shape and contained fewer, as well as smaller, LDs (Fig. 4B). As shown in Fig. 3C,D, BBR as well as *C. chinensis* inhibited adipogenic differentiation in a concentration dependent manner, with a maximum effect detected at a concentration of 1.1 µg/mL (BBR, p > 0.0001) and 5 mg/L (*C. chinensis*, p < 0.0001). In contrast, lipid accumulation was unaffected when mature 3T3-L1 cells were treated (Figs 3A and 4A), except for *C. chinensis* (p < 0.01), indicating the remarkable anti-adipogenic properties of this extract.

### Effects of BBR and herbal extracts on intracellular lipid content

**Figure 5:**
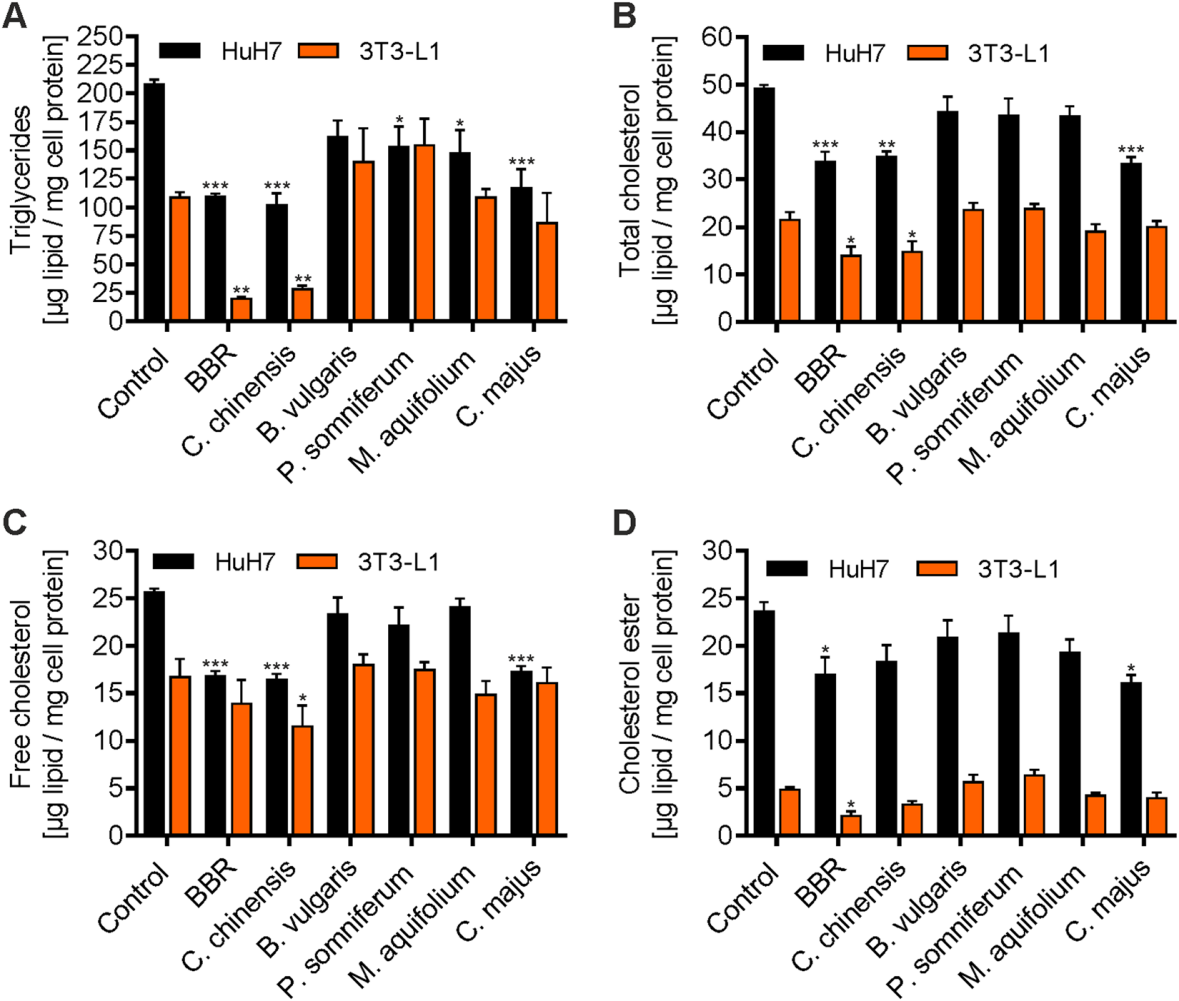
Effects of BBR and herbal extracts on triglycerides and on cholesterol. Cells were treated with BBR and the indicated herbal extracts, followed by lipid extraction and direct quantitation using GC for (**A**) triglycerides, (**B**) total cholesterol, (**C**) free cholesterol, and (**D**) cholesterol ester. Error bars are based on the SEM of at least two experiments. *p < 0.05, **p < 0.01 and ***p < 0.001, significantly reduced lipid content compared to untreated control cells.

We investigated the effects of herbal extracts on the intracellular content of neutral lipids and cholesterol in HuH7 and 3T3-L1 pre-adipocytes (Fig. 5). For this purpose, the quantitative analysis of triglycerides (TGs, Fig. 5A), total cholesterol (TC, Fig. 5B), free cholesterol (FC, Fig. 5C) and cholesterol esters (CE, Fig. 5D) was performed using gas chromatography. Generally, similar results were obtained for both HuH7 and 3T3-L1 cells. In summary, treatment with BBR and *C. chinensis* significantly reduced TG, TC, FC and CE accumulation when compared to untreated cells. Noteworthy, *C. majus* treatment led to a significant reduction in triglycerides and cholesterol in HuH7 cells but not in 3T3-L1 cells.

### Effects of BBR and herbal extracts on isoproterenol-stimulated lipolysis

**Figure 6:**
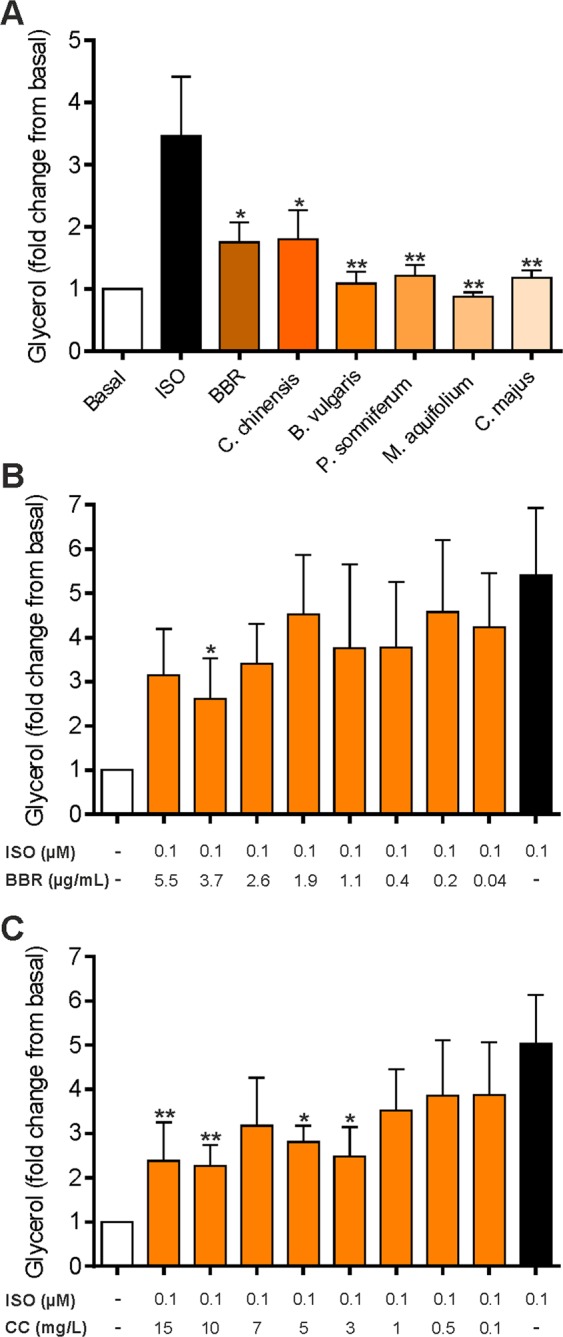
Effects of BBR and herbal extracts on isoproterenol-stimulated lipolysis in 3T3-L1 adipocytes. Differentiated 3T3-L1 cells were incubated with BBR and herbal extracts for 24 hours and subsequently stimulated with 100 nM isoproterenol an additional hour. The medium was transferred into a new 96-well plate for glycerol measurement on the plate reader. (**A**) 3T3-L1 adipocytes were incubated with BBR and various extracts for 24 hours prior to isoproterenol (ISO) stimulation. (**B**) 3T3-L1 adipocytes were incubated with various concentrations of BBR for 24 hours. (**C**) 3T3-L1 adipocytes were incubated with various concentrations of *C. chinensis* (CC) for 24 hours. Error bars are based on the SEM of at least three experiments. *p < 0.05, **p < 0.01, significantly reduced glycerol content compared to isoproterenol-treated cells.

BBR has been reported to attenuate lipolysis stimulated by catecholamines^41^. In order to investigate the role of the herbal extracts used in this study within this context, differentiated 3T3-L1 cells were preincubated with indicated substances for 24 hours followed by additional stimulation with isoproterenol for further 1 hour (Fig. 6A). Cells incubated with isoproterenol showed a ~3.5-fold increase in glycerol release, which was suppressed ~1.8-fold by BBR and *C. chinensis* (p < 0.05) and ~2.5-fold by *B. vulgaris*, *P. somniferum*, *M. aquifolium* and *C. majus* (p < 0.01). To test for a concentration-dependent decrease in isoproterenol-stimulated lipolysis, cells were treated with different concentrations of BBR and *C. chinensis*. As shown in Fig. 6B,C, BBR and *C. chinensis* significantly decreased lipolysis with a maximal effect at the concentration of 3.7 µg/mL (BBR, p < 0.05) and 10 mg/L (*C. chinensis*, p < 0.01), respectively.

### Effects of BBR and herbal extracts on fatty acid uptake

**Figure 7:**
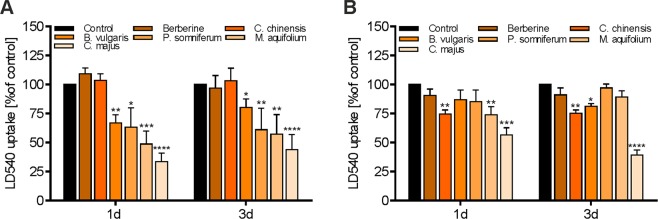
Effects of BBR and herbal extracts on fatty acid uptake in HuH7 and 3T3-L1 adipocytes. Treated cells were pulse labeled for 20 s with LD540 (0.5 µg/mL) and further incubated in label-free medium for 30 min. Afterwards, cells were lysed with 0.05 M NaOH and LD540 accumulation was measured on a plate reader. (**A**) Effects of BBR and herbal extracts on LD540 uptake in 3T3-L1 adipocytes treated for 1 and 3 days with the indicated substances post-differentiation. (**B**) Effects of BBR and herbal extracts on LD540 uptake in HuH7 cells treated for 1 and 3 days with the indicated substances. Error bars are based on the SEM of three experiments. *p < 0.05, **p < 0.01, ***p < 0.001 and ****p < 0.0001, significantly reduced fatty acid uptake of cells treated with indicated substances compared to untreated control cells.

Most recently, BBR has been shown to enhance fatty acid uptake and lipid accumulation by increasing the expression and membrane translocation of CD36 (fatty acid transporter) in mouse hepatocytes^42^. To investigate the effects of BBR and the herbal extracts used in this study, fatty acid transport in differentiated 3T3-L1 and HuH7 cells was analyzed using the fluorescently labeled fatty acid tracer LD540. 3T3-L1 adipocytes and HuH7 cells were pre-treated with the indicated substances for 1 and 3 days, subsequently incubated with LD540, and fluorescence intensity was measured (Fig. 7). The treatment of 3T3-L1 adipocytes with BBR and *C. chinensis* did not significantly influence LD540 uptake, except for a slight increase after 1 day, when compared to untreated cells (Fig. 7A). This result indicates that the hypolipidemic effects of BBR cannot be attributed to reduced lipid uptake. Interestingly, the herbal extracts with only minor or not quantifiable amounts of BBR significantly reduced LD540 uptake after 1 day of treatment, with *C. majus* tending to be the most potent inhibitory extract (p < 0.0001 after 1 and 3 days), followed by *M. aquifolium* and *P. somniferum*. Similar results were obtained in HuH7 cells (Fig. 7B), with *C. majus* again being the most potent LD540 uptake inhibitor (p < 0.001 and p < 0.0001 after 1 and 3 days, respectively).

### Effects of herbal extracts on intracellular fatty acid exchange

**Figure 8:**
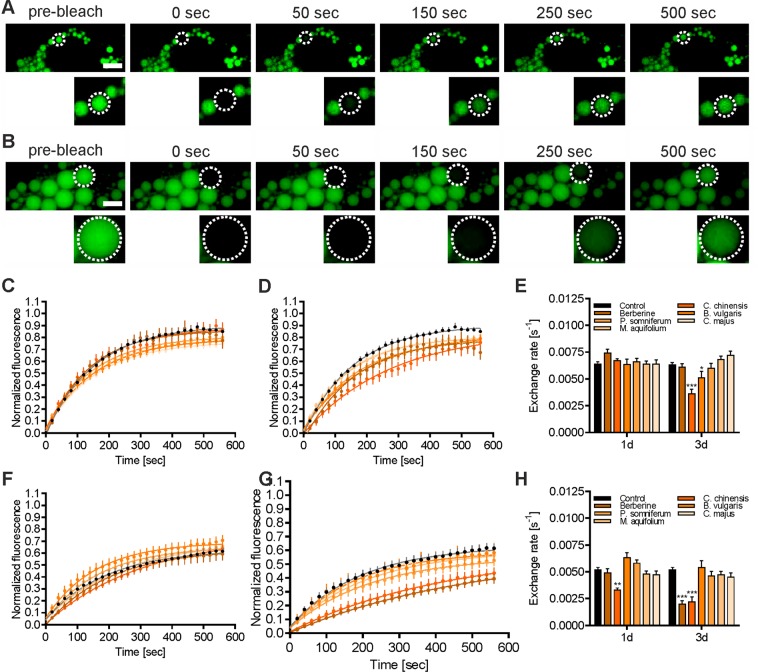
Kinetics of LD540 transport for different LD size fractions. 3T3-L1 adipocytes were pretreated with the indicated substances for 1 or 3 days post differentiation or left untreated (control cells), labeled with LD540 for 5 minutes, rinsed, and chased without labeling for an additional 30 min. Single LDs were bleached with an intense laser pulse (405 nm, 1 sec) and fluorescence recovery of the bleached LD was detected for 10 minutes, using confocal microscopy. LDs were subdivided into small (3–6 µm diameter) and large (8–12 µm diameter) fractions. Representative fluorescence images of cells with small (**A**) or large (**B**) LDs are shown at the indicated time points before and after photobleaching. Scale bar = 10 µm. Normalized mean fluorescence recovery curves of bleached small LDs pretreated for 1 and 3 days post differentiation with the indicated extracts are shown in (**C**,**D**), respectively. (**E**) Calculated exchange rates from a single exponential fit of fluorescence traces are shown in (**C**,**D**). Normalized mean fluorescence recovery curves of bleached large LDs pretreated for 1 and 3 days post differentiation with the indicated extracts are shown in (**F**,**G**), respectively. (**H**) Calculated exchange rates from a single exponential fit of fluorescence traces are shown in (**F**,**G**). Error bars are based on the SEM of at least 19 analyzed cells from three individual experiments. *p < 0.05, **p < 0.01, ***p < 0.001, and ****p < 0.0001, significantly reduced exchange rate of cells treated with the indicated substances compared to untreated control cells.

Lipid biosynthesis and lipolysis exhibit highly compartmentalized processes in adipocytes^43–46^, suggesting the transient regulation of lipid stores. It has been recently shown that intracellular lipids continuously exit and re-enter LDs and that the LD size determines transport efficiency^47^. To investigate the impact of herbal extracts on intracellular lipid traffic in differentiated 3T3-L1 adipocytes, we employed live-cell confocal fluorescence microscopy, in combination with the fluorescently labeled fatty acid tracer LD540. To quantitate the intracellular fatty acid mobility, we utilized the fluorescence recovery after photobleaching (FRAP) technique. Selected LDs of different sizes (subdivided into small (3–6 µm diameter) and large (8–12 µm diameter) LDs)^47^ were then photobleached by targeted laser illumination, and changes in the fluorescence of the photobleached LDs were monitored over time (Fig. 8A,B). Based on the analysis of the kinetics of LD540 exchange in small LDs (Fig. 8C,D), the pretreatment of adipocytes with the indicated herbal extracts for 1 day did not influence the rate of fluorescence recovery, whereas significant reductions in the exchange rate were detected after 3 days of pretreatment with *C. chinensis* (p < 0.001) and *B. vulgaris* (p < 0.05), compared to control cells (Fig. 8E). In contrast, the kinetics of LD540 exchange in large LDs (Fig. 8F,G) was significantly reduced after 1 day of *C. chinensis* treatment (p < 0.01) (Fig. 8H). The exchange rate was further decreased after 3 days of pretreatment (p < 0.001), and a similar reduction potential was detected for BBR (p < 0.001).

### Effects of herbal extracts on LD motility

**Figure 9:**
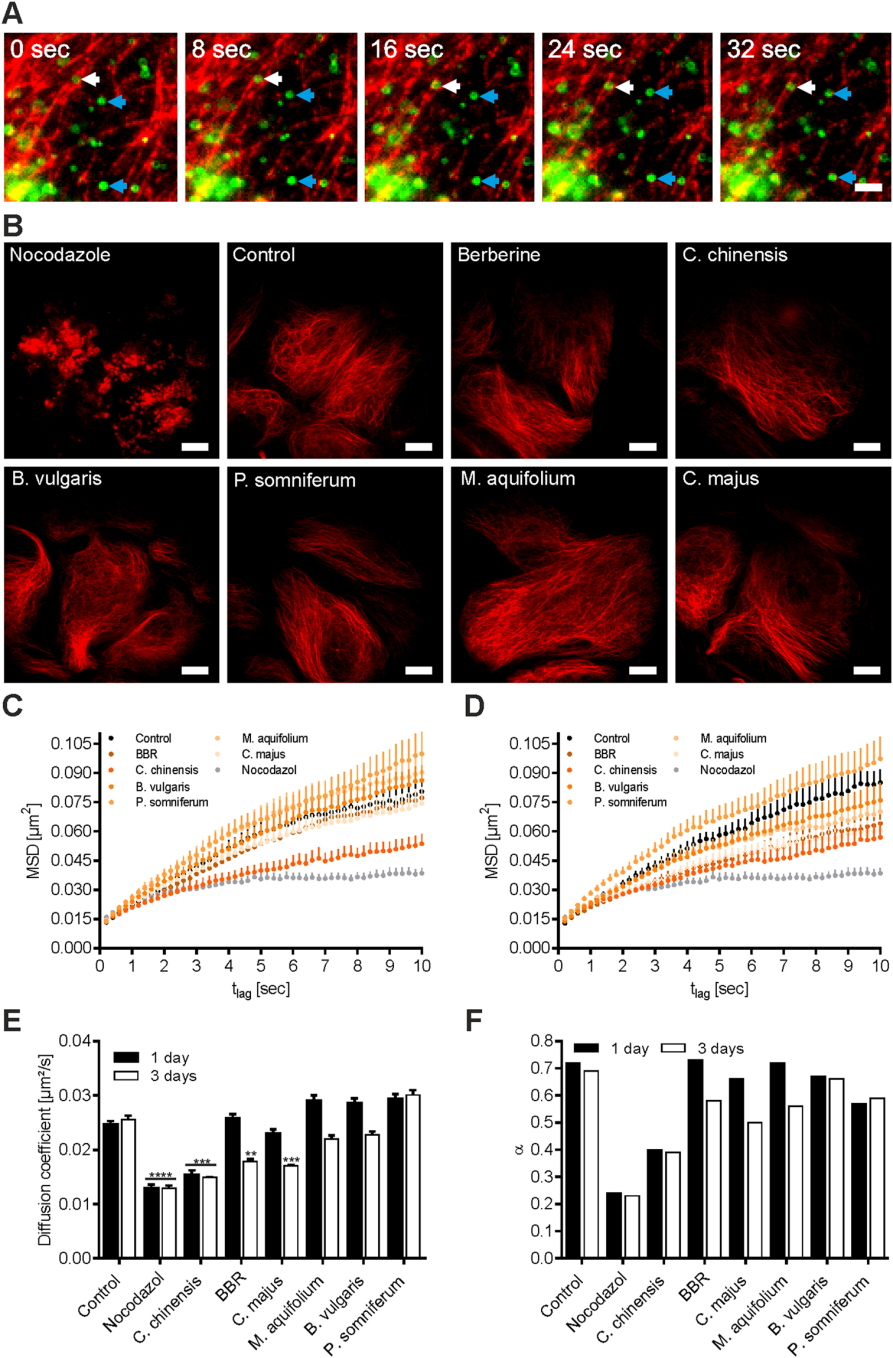
LD motility tracking. (**A**) Movement of LDs in HuH7 cells. HuH7-ADRP-GFP cells (green) were stained with SiR-tubulin (red) and the movement of LDs was imaged via TIRF microscopy by tracking the ADRP-GFP signal. Most of the LDs are oscillating or stationary (blue arrows), while some move along microtubules (white arrow). Scale bar = 15 µm. (**B**) Representative confocal images of HuH7-ADRP-GFP cells treated with nocodazole (2 µg/mL for 1 hour) or the indicated substances for 3 days and subsequently stained with SiR-tubulin. Scale bar = 10 µm. (**C**) Average mean square displacements (MDS) of LDs in HuH7-ADRP-GFP cells as a function of lag time after being pretreated for 1 day with the indicated substance or left untreated (control). (**D**) Average mean square displacements (MDS) of LDs in HuH7-ADRP-GFP cells as a function of lag time after being pretreated for 3 days with the indicated substance or left untreated (control). MSD plots are based on at least 100 analyzed cells ± SEM. (**E**) Diffusion coefficients for the indicated treatment within the linear range were determined by fitting the function MSD = 4Dt_lag_ + 4dx^2^. Error bars are based on the SEM with **p < 0.01, ***p < 0.001, and ****p < 0.0001, significantly reduced D of cells treated with the indicated substances compared to untreated control cells. (**F**) α-values for the indicated treatments were obtained by fitting the sub-linear (anomalous diffusion) function MSD = 4Dt_lag_^α^ + 4dx². Respective linear and sub-linear fits were not illustrated to retain clearly represented data and figures.

LDs show both microtubule-based, directional, long-distance and random, short-distance (oscillating) movements, and active LD movement is related to the regulation of the LD’s intracellular distribution and interactions with other cell organelles^17,48^. Furthermore, it has been speculated that active movement along microtubules promotes the formation of larger LDs, which are presumably more resistant to lipolysis than smaller LDs^17^. HuH7 cells stably expressing the GFP-labeled adipocyte differentiation-related protein (ADRP-GFP) were used to investigate the effects of herbal extracts on LD motility. ADRP is an ubiquitous component of LDs^49,50^ and, therefore, offers the potential to analyze the behavior of LDs in live cells when tagged with fluorescent proteins^51^. Cells were stained with SiR-tubulin, and live-cell confocal fluorescence microscopy was used to observe microtubule-based, long-distance (Fig. 8A, white arrow) and oscillating (Fig. 9A, blue arrows) LD movements. Pretreatment with nocodazole (2 µg/mL for 1 hour), a known agent that interferes with the polymerization of microtubules^52^, significantly reduced SiR-tubulin incorporation as a result of reduced microtubule formation (Fig. 9B). To exclude the possibility that any observed changes in LD motility caused by treatment with BBR or herbal extracts was due to microtubule destruction, cells were treated for up to 5 days with the indicated substances or left untreated (control cells) and were subsequently stained with SiR-tubulin and imaged by live-cell confocal microscopy. As shown in Fig. 8B, treatments with BBR and herbal extracts did not have any negative effects on microtubule formation and appearance. To examine the possible effects of herbal extracts on LD transport mechanisms, a mean square displacement (MSD) analysis was performed (Fig. 9C,D). For this purpose, cells were pretreated for 1 (Fig. 9C) or 3 (Fig. 9D) days with the indicated substances or left untreated (control), and LD motion was subsequently imaged by collecting the ADRP-GFP emissions during a period of 10 sec, with a time lag of 200 ms. In general, the observed droplet movements could be categorized into two different groups: 1) free Brownian motion, characterized by a linear increase in MSDs with a time-lag (~0–3 sec) and, 2) a sub-linear increase in MSDs, in which droplets showed anomalous subdiffusion (~0–10 sec). Nocodazole was again used as a negative control, as this treatment causes the destruction of the microtubule network but leaves the actin cytoskeleton of the cell intact. For nocodazole treated cells (2 µg/mL for 1 hour), LDs were observed to move with fewer long range directed displacements when compared to untreated cells (Fig. 9C,D), resulting in a significantly reduced linear diffusion coefficient (D) (p < 0.0001, with D = 0.0129 ± 0.0020 µm²/s for nocodazole and D = 0.0247 ± 0.0013 µm²/s for untreated cells) (Fig. 9E). Similar results were obtained for cells treated with *C. chinensis* extract for 1 day (p < 0.001, with D = 0.0154 ± 0.0018 µm²/s). A prolonged cell treatment (3 days) led to further significantly reduced MSD (Fig. 9D,E) values for BBR (p < 0.01, with D = 0.0178 ± 0.0011 µm²/s) and *C. majus* (p < 0.001, with D = 0.0171 ± 0.0011 µm²/s). A comparable trend was observed for the characterization of the time range of sub-diffusive motion (~3–10 sec), with the smallest anomalous diffusion exponent for nocodazole (α ~ 0.24) and *C. chinensis* (α ~ 0.40) treated cells (Fig. 9F), indicating the increased confinement of LDs.

## Discussion

Plant derived products and their bioactive compounds have played an inherent role in human nutrition and medicine for many years. Due to the severe side effects of conventional drugs used in the treatment of obesity, medicinal plants, herbal extracts and isolated phytochemicals have drawn attention because of their relative safety, low cost and the accumulating evidence of physiological properties such as anti-adipogenic effects^53–55^. In this context, herbal extracts containing large amounts of flavonoids, stilbenoids, phenolic acids and alkaloids have been extensively investigated^38^.

Berberine and other related plant alkaloids such as epiberberine, coptisine, palmatine, magnoflorine, bouchardatine, trigonelline, antofine and evodiamine, have been reported to affect adipogenesis at low concentrations^38,40,56–60^. We therefore aimed to investigate putative alkaloid-rich herbal extracts, obtained from the open access screening library PECKISH^24^. Herbal extracts from Chinese goldthread roots (*C. chinensis rad*.), barberry stem barks (*B. vulgaris cortex lign*.), opium poppy capsules (*P. somniferum capsula*), mahonia roots (*M. aquifolium rad*.) and tetterwort plants (*Chelidonium majus herb*.) were selected, and the BBR content was quantitated as the main bioactive lead-alkaloid by HPLC analysis (Fig. 1 and Table 1). Interestingly, herbal extracts exhibited large variations in BBR content. The extract of *C. chinensis* was found to contain the highest BBR content (2.78 g/L), followed by *M. aquifolium* (0.03 g/L) and *B. vulgaris* (0.02 g/L). No quantifiable BBR content was detected in the extracts of *C. majus* and *P. somniferum*. Nevertheless, *C. majus* turned out to be a potent herbal extract, as treatment led to slightly reduced adipocyte differentiation (Fig. 3B) and a significant reduction in glycerol release (Fig. 6A) and intracellular fatty acid uptake (Fig. 7), with a subsequent reduction in intracellular neutral lipid content (Fig. 5), indicating phytogenic activity independent of BBR. Furthermore, *C. majus* significantly reduced LD motility after 3 days of pretreatment (Fig. 9D,E). We speculate that the observed effects might be associated with further identified bioactive alkaloids such as magnoflorine, chelidonine, coptisine and the BBR-derivative palmatine. However, we cannot exclude the presence of other, currently unknown phytochemicals that could account in addition and lead to different mode of action. A direct chemical comparison against other reported extracts is challenging, as differences in the BBR and alkaloid contents of the studied herbal extracts underlie large differences with respect to different plant species, parts and extraction procedures^61–66^. Furthermore, no detailed information regarding extract preparation is available from the PECKISH library.

Increases in the number and size of adipocytes (differentiated from pre-adipocytes) has been reported to cause the excessive growth of adipose tissue mass, subsequently leading to obesity^67,68^. Our results show that BBR and a BBR-rich extract from *C. chinensis* significantly inhibited the viability of differentiated 3T3-L1 cells after long-term treatment (5 days, Fig. 2C). They also remarkably suppressed adipogenic differentiation, as indicated by the presence of fewer mature adipocytes with LDs (Fig. 4), presumably caused by the down-regulation of the expression and activity of important adipocyte-specific genes (such as C/EBPα, C/EBPβ, PPARγ2, SREBP-1c, FAS, ACC, and ACS), as has been previously reported for BBR and other plant alkaloids^10,14,38,40,55–58^. Furthermore, BBR and *C. chinensis* reduced the level of accumulated intracellular neutral lipids (Fig. 5). Interestingly, the extract of *C. majus* (containing no quantifiable amount of BBR), also slightly reduced adipogenic differentiation and led to a strong reduction in neutral lipids, indicating a BBR-independent effect. *C. majus* herbs have been reported to be enriched in various alkaloids (protopine, chelidonine, coptisine, stylopine, sanguinarine, berberine and chelerythrine)^69,70^, which might account for the observed effects. In the present extract we could at least identify chelidonine, magnoflorine, coptisine and the BBR-derivative palmatine. However, other bioactive phytochemicals cannot be excluded and must be investigated in the future. Taken together, these findings suggested that BBR, *C. chinensis* and *C. maju*s can play inhibitory roles in the conversion of 3T3-L1 cells during adipogenesis, apart from their inhibitory effects on cell viability. The activation of AMP-activated protein kinase (AMPK) mimics a lack of cellular energy and decreases energy-consuming processes, such as lipid synthesis. Importantly, BBR was found to inhibit fatty acid and cholesterol synthesis through the activation of AMPK^71^. The combined effects of BBR and BBR-containing herbal extracts on triglyceride and cholesterol levels (Fig. 5) might thus be a consequence of reduced lipid synthesis.

Synthetic catecholamines such as isoproterenol have been shown to stimulate lipolysis primarily via cAMP-mediated activation of protein kinase A (PKA) and ERK 1/2^72^. BBR has already been reported to directly decrease catecholamine-stimulated lipolysis mainly by reducing the inhibition of phosphodiesterase (PDE), leading to a decrease in cAMP and hormone-sensitive lipase (HSL) phosphorylation, independent of the AMPK pathway^41^. A comparable effect was observed in our experiment for BBR-treated 3T3-L1 adipocytes (Fig. 6), confirming the antilipolytic property of BBR. Therefore, lipolysis does not seem to be causally involved in the reduction of triglyceride levels observed after BBR treatment. Rather, reduced lipolysis might be a consequence of low triglyceride stores. Regarding the other herbal extracts tested in our study, no data on the regulation of lipolysis are available from the literature. Interestingly, all of the putative alkaloid-rich extracts exhibited significant inhibition of isoproterenol-induced stimulation of lipolysis, indicating BBR-independent mechanisms and other bioactive constituents.

Most recently, various isoquinoline alkaloids, including BBR, sanguinarine and aromoline, were shown to modulate intracellular lipid accumulation through AMPK activation and the subsequent translocation of the CD36 fatty acid transporter to the cell membrane, mediating lipid uptake^42,73,74^. However, not much is known regarding the effects of herbal extracts used in this study on this process. Surprisingly, several herbal extracts have been revealed to be potent fatty acid uptake inhibitors, as assessed by LD540 uptake studies (Fig. 7). In 3T3-L1 adipocytes, the extracts of *B. vulgaris*, *P. somniferum*, *M. aquifolium* and *C. majus* significantly reduced intracellular LD540 uptake. BBR and *C. chinensis*, as expected, led to a slightly increased uptake after 1 day of treatment and did not affect LD540 uptake after 3 days. Similar results were obtained in HuH7 cells, with *C. majus* being the most potent inhibitor. However, in HuH7 cells, *C. chinensis* was also found to significantly reduce LD540 uptake, which might indicate different regulatory mechanisms in various tissues. High circulating levels of free fatty acids with subsequently elevated internalization have been reported to exceed the intracellular triglyceride storage capacity, resulting in high levels of fatty acid oxidation, oxidative stress and ceramide production^75,76^. This process is called lipotoxicity and is hypothesized to correlate with the development of obesity and related diseases^77^. Therefore, in addition to the inhibition of adipogenesis, the regulation of fatty acid internalization could be another potential strategy to prevent and treat obesity^78^. Further investigations are required to study the detailed regulatory mechanisms and to identify the putative bioactive phytochemicals in our herbal extracts.

Neutral lipids are accumulated and stored into LDs in the cytosol of many different cells. Despite variations in size (sub-µm to 200 µm in diameter) and appearance, the structure and organization of LDs is highly conserved^79^. However, the functional implications of the different LD size fractions are poorly understood. However, there is recent evidence that large LDs (7–10 µm diameter) are less efficient at transporting and, possibly, metabolizing fatty acids than small LDs (2–5 µm diameter), indicating that small LDs may function as highly dynamic and metabolically active organelles^47^. Here, we used FRAP to study the impact of BBR and herbal extracts on cellular lipid flow and lipid exchange between LDs and the cytosol (Fig. 8). We could unequivocally show that BBR and *C. chinensis* reduced the intracellular fatty acid (LD540) mobility, and that these effects were more prominent in large LDs (8–12 µm diameter) than in small LDs (3–6 µm diameter). The conversion of fatty acids into triacylglycerol (TAG) and the subsequent uptake of TAG into LDs is regulated by a series of enzymatic reactions, catalyzed by glycerol-3-phosphate O-acyltransferase (GPAT), 1-acylglycerol-3-phosphate O-acyltransferase (AGPAT), phosphatidic acid phosphatase (PAP)/lipin, and diglyceride acyltransferase (DGAT)^80^. TAG is generated at the endoplasmic reticulum (where most of the enzymes are located) and transported to LDs through unknown mechanisms^81^. In addition, various enzymes have been reported to be localized to LDs and to promote local TAG production and LD growth^80^. To the best of our knowledge, this is the first demonstration that phytochemicals specifically regulate the fatty acid/TAG mobility and incorporation into LDs, dependent of LD size. However, the influence on specific regulatory mechanisms must still be unraveled, as well as the functional consequences of reduced lipid exchange.

LD movement is thought to regulate LD growth and promote the formation of larger, more lipolysis-resistant LDs^17^. Therefore, enlarged intracellular triglyceride repositories might contribute to the development of obesity. Knowledge regarding cellular LD movement and the possible effects of extracellular agents, such as phytogenic substances, might be of pivotal importance for developing novel anti-obesity strategies in the future. In general, in our HuH7 cell model, most LDs showed oscillating movement within a confined area, and only a small number of LDs could move rapidly, indicating the transient association with intracellular transport pathways (Fig. 9A). This observation is in accordance with a previous study that also used HuH7 cells stably expressing GFP-tagged ADRP^51^. Live cell LD tracking and MSD analysis revealed free diffusion for time-lags <3 sec, as well as anomalous sub-diffusive motion in HuH7 cells for longer time-lags (3–10 sec) (Fig. 9C,D). Subdiffusion is characterized by MSDs obeying a power law at exponents <1 (MSD ~ t^α^, α < 1)^82^, whereas the reasons for confined motion are manifold, including crowding, stalling, obstruction barriers and trapping cages^82–84^. Anomalous diffusion in the cytoplasm of living cells has been reported for viruses^85^, telomers^86^, lipid granules^87^, and LDs^88^. We could unequivocally show that phytogenic substances effect LD movement in live cells. Interestingly, *C. chinensis* and *C. majus* extract, as well as BBR treatment, led to similar reductions in LD movement as observed in nocodazole treated cells but without affecting the microtubule network (Fig. 9B), indicating different points of action. In nocodazole-treated cells, LDs could be tracked for longer time periods, as fewer left the field of view, indicating a high degree of confinement, most likely caused by the absence of the microtubule network. However, there might be additional reasons for a more confined LD behavior upon herbal extract treatment, such as (i) the inhibition of LD motor proteins, such as dynein and kinesin-1, (ii) changes in the regulatory droplet-localized proteins, such as PAT proteins (perilipin/ADRP), (iii) changes in cell size and volume, (iv) a more “crowded” cytosolic environment due to the prevention of large LD formation, and (v) altered cytosolic neutral lipid content.

In summary, our findings suggest that potential alkaloid-rich herbal extracts are able to influence important mechanisms regulating adipocyte differentiation, lipid synthesis, storage and mobilization. To the best of our knowledge, this is the first study to demonstrate that phytogenic substances can influence the kinetics of TAG mobility and incorporation in LDs, as well as LD motility.

## Data Availability

The datasets generated during and/or analyzed during the current study are available from the corresponding author upon reasonable request.
